# Smartphone-Based Solutions for Fall Detection and Prevention: Challenges and Open Issues

**DOI:** 10.3390/s140407181

**Published:** 2014-04-22

**Authors:** Mohammad Ashfak Habib, Mas S. Mohktar, Shahrul Bahyah Kamaruzzaman, Kheng Seang Lim, Tan Maw Pin, Fatimah Ibrahim

**Affiliations:** 1 Department of Biomedical Engineering, Faculty of Engineering, University of Malaya, 50603 Kuala Lumpur, Malaysia; E-Mails: ashfak@siswa.um.edu.my (M.A.H.); mas_dayana@um.edu.my (M.S.M.); 2 Centre for Innovation in Medical Engineering (CIME), Faculty of Engineering, University of Malaya, 50603 Kuala Lumpur, Malaysia; E-Mails: shahrulk@um.edu.my (S.B.K.); kslimum@um.edu.my (K.S.L.); mawpin@um.edu.my (T.M.P.); 3 Department of Computer Science & Engineering, Chittagong University of Engineering & Technology, Chittagong 4349, Bangladesh; 4 Department of Medicine, Faculty of Medicine, University of Malaya, 50603 Kuala Lumpur, Malaysia

**Keywords:** fall detection, fall prevention, smartphone, ubiquitous computing, pervasive computing, elderly

## Abstract

This paper presents a state-of-the-art survey of smartphone (SP)-based solutions for fall detection and prevention. Falls are considered as major health hazards for both the elderly and people with neurodegenerative diseases. To mitigate the adverse consequences of falling, a great deal of research has been conducted, mainly focused on two different approaches, namely, fall detection and fall prevention. Required hardware for both fall detection and prevention are also available in SPs. Consequently, researchers' interest in finding SP-based solutions has increased dramatically over recent years. To the best of our knowledge, there has been no published review on SP-based fall detection and prevention. Thus in this paper, we present the taxonomy for SP-based fall detection and prevention solutions and systematic comparisons of existing studies. We have also identified three challenges and three open issues for future research, after reviewing the existing articles. Our time series analysis demonstrates a trend towards the integration of external sensing units with SPs for improvement in usability of the systems.

## 1. Introduction

Falls are defined as the inadvertent settling down of a body on the ground, floor or other lower level. The prevalence of falls is very common among the elderly and increases with age. The World Health Organization (WHO) reported that 28%–35% of people aged 65 years and above fall each year and the rate increases to 32%–42% for those over 70 years of age [1]. Those who are vulnerable to falls also include those suffering from neurological diseases (e.g., epilepsy and dementia), which commonly occur in older people. Individuals with epilepsy fall during seizure events due to loss of consciousness [2], while those with dementia are two to three times more likely to fall than individuals without cognitive impairment [3]. Living alone itself increases the risk of falls for community elders [4]. Falls can potentially cause severe physical injuries such as disabling fractures [5], and can reduce the independence of older individuals through dramatic psychological consequences [6]. If protective measures cannot be taken in the near future, the number of falls induced injuries is anticipated to double by 2030 [7].

Hence, early detection and treatment of falls are key strategies to be employed in reducing fall- related injuries and preventing their consequences, which include long laying periods (remaining on the floor for prolonged periods after a fall) leading to an increased risk of pneumonias, pressure ulcers and even death. The use of assistive devices for fall detection and prevention will help reduce its future burdens by preventing injurious falls, reducing the risk of long laying periods and admissions to nursing homes. Insights gained from research in this area by industry and academics will assist community, public health leaders and health care professionals in developing more efficacious intervention strategies to prevent or reduce falls, and its associated psychological, physical and economical consequences.

This past decade alone has seen a tremendous amount of research in the development of assistive devices for fall management. Researchers and industry mainly focus on two automatic fall management strategies namely, its detection and prevention. Typically fall detection systems help the elderly and their caregivers avoid the consequences of long laying periods by detecting falls, triggering notification alarms, sending messages and calling for help as soon as falls occur. Fall prevention systems are usually based on the assessment of the medical and behavioral histories of users in order to predict the possible risk of falls. Most of these fall management technologies consist of three common functional units: a sensing/data-acquisition unit, processing unit and communication unit. The accelerometer, gyroscope and camera are the most frequently used sensors in SPs, while Bluetooth and Wireless Fidelity (Wi-Fi) technologies are typically used for communication purposes. Various microcontrollers and wirelessly connected desktops or laptops are usually used for feature extraction and classification from the sensors' output signals. SP-based fall detection and prevention is attracting growing interest among researchers as state-of-the-art SPs come with built-in kinematic sensors (such as tri-axis accelerometers, gyroscopes, and magnetic sensors), high performance microprocessors, advance communication facilities (e.g., Wi-Fi and Bluetooth) and other sensors (such as camera, proximity sensor and microphone) [8]. In a recent survey, Igual *et al.* [7] have shown a new trend towards the integration of fall detection into SPs.

A variety of dedicated tools and methods have been proposed for fall management, but none of these solutions is universally accepted [9]. The SP however, is a very good candidate as this technology is widely accepted in daily life [10]. SPs are also more integrated than a dedicated monitoring device which reduces rejection due to the device's poor aesthetic value and intrusiveness [11]. For these and many other reasons, the number of studies on SP-based fall management has increased steadily in recent years. Currently, to the best of our knowledge, there has been no published review specifically on SP-based fall detection and prevention systems. Although, there are some relevant review articles [7,12,13], there are none that focus exclusively on SP-based fall detection and prevention systems.

This paper provides a comprehensive and integrative literature review of SP-based fall detection and prevention systems. The usability and overview of the general architecture of SP for fall management with several new dimensions including a comprehensive taxonomy of the SP-based fall management systems is presented. A critical analysis of the methods proposed so far and a comparison of their features, strengths and weaknesses is made. This includes the identification of the issues and challenges found with the SP-based fall management systems.

Throughout this paper, the terms *fall prediction* and *fall prevention* are used interchangeably because SP-based fall prevention systems attempt to prevent falls by predicting the imminent fall events. Unless otherwise stated, accelerometer and gyroscope represent tri-axial-accelerometer and tri-axial-gyroscope respectively. A SP is a combination of a normal mobile phone and a Personal Digital Assistant (PDA) [14]. Ordinary mobile phones and PDAs have less functionality than SPs and cannot be considered as SPs. Therefore, PDA or pocket Personal Computer (PC)-based [15,16] and ordinary mobile phone-based [17] solutions are excluded from our comparative study.

This paper is organized in five sections: Section 2 discusses the basic architecture and taxonomy of SP-based fall detection and prevention systems. A comparative analysis of the reviewed articles is provided in Section 3, illustrated by tables and graphs. Section 4 highlights the challenges of the SP- based solutions and also discusses some open issues. Finally, the concluding part—Section 5—points out important observations and areas that need further research.

## 2. SP Based Fall Detection and Prevention

Although a fall detection system was first introduced by Hormann in the early 1970s [18,19], the history of SP-based fall detection is far shorter. The first smartphone (“Simon”) was first introduced by IBM in 1993 [20] and subsequently, various sensors useful for human activity monitoring were integrated into SPs. Hansen *et al.* [21] used the SP camera for the first time in 2005 for fall detection. The SP is also used for fall prevention [22], but instead of active fall prevention, most of the solutions proposed were based on standard falls risk assessment tests Timed Up and Go (TUG) and Get Up and Go (GUG).

### 2.1. Basic Architecture

**Figure 1.**
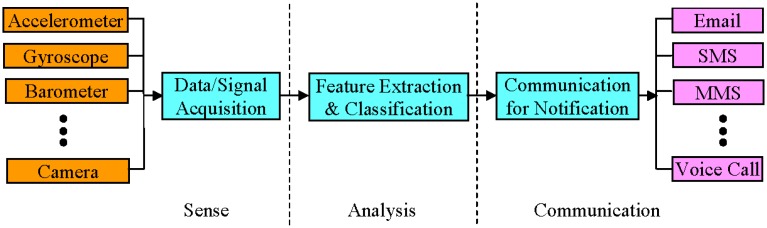
Common basic architecture of fall detection and fall prevention systems.

Fall detection and fall prevention systems have the same basic architecture as shown in Figure 1. Both systems follow three common phases of operation: sense, analysis and communication. The basic difference between the two systems lies in their analysis phase with differences in their feature extraction and classification algorithms. Fall detection systems try to detect the occurrence of fall events accurately by extracting the features from the acquired output signal(s)/data of the sensor(s) and then identifying fall events from other activities of daily living (ADL). On the other hand, fall prevention systems attempt to predict fall events early by analysing the outputs of the sensors. Data/signal acquisition, feature extraction and classification, and communication for notification are the necessary steps needed for both fall detection and prevention systems. The number and type of sensors and notification techniques however, vary from system to system (some examples are shown in Figure 1). In conventional systems, discrete hardware components are used for the implementation of each unit, whereas in SP-based systems, all required units may already be in-built within a state-of-the-art SP.

#### 2.1.1. Phase 1: Sense

This is the first phase of any fall detection and prevention system and in this phase, appropriate physical quantities are sensed or measured using suitable sensors. Modern SPs come with various built-in sensors and that is one of the vital reasons for choosing SPs as an alternative of conventional fall detection and prevention tools [9]. Moreover, the users of SP-based systems are more likely to carry SP (with built-in sensors) throughout the day since mobile phones are seen as indispensable in daily living. This is in contrast to the users of the conventional systems who may forget to wear the special microsensors [17]. Many types of sensors are now available for SPs. These include accelerometers, gyroscopes, temperature sensors and magnetic field sensors [23–25]. These sensors are used in various ways in SP-based solutions. Some solutions use only one of the abovementioned SP sensors for fall detection or prediction [26,27]. According to our survey, the tri-axial accelerometer is the most used sensor for SP-based fall detection and prevention. SP-based solutions can use combinations of two or more SP sensors during this sensing phase [22,28]. Some solutions use both SP sensors and external sensors for detection and prediction of falls events [29,30]. It is also possible to use SPs for analysis and/or communication but not for sensing [31,32]. An uncommon type of solution was proposed by Hansen *et al.* [21]. They used a SP for sensing only, and external systems to perform the analysis and communication tasks.

#### 2.1.2. Phase 2: Analysis

After measuring the physical quantities by using sensors, obtained signals/data should be analysed. In this phase, the significant features are extracted from the sensor's outputs and preliminary decisions are made by classifying and analysing those extracted features. Most SP-based solutions, especially solutions for fall detection, use a Threshold-Based Algorithm (TBA). The most vital reason for choosing TBAs is that these algorithms are less complex and hence require the lowest computational power [9], which helps to reduce battery power consumption [33]. In order to make preliminary decisions about a potential fall event, these algorithms usually compare the sensor's output(s) with predefined threshold value(s). Threshold-based algorithms may use more than one threshold [27] and threshold value(s) could be predefined (fixed) or adaptive. It should be noted that the adaptive threshold values are not calculated dynamically while using the system. Instead, users introduce some physiological data and the system obtains the corresponding threshold that is not re-calculated during the system operation. The algorithm proposed in [34] uses an adaptive threshold which changes with user-provided parameters such as: height, weight and level of activity.

As mentioned earlier, most solutions employ the tri-axial accelerometer for sensing which measure simultaneous accelerations in three orthogonal directions. Threshold-based algorithms use these acceleration values for calculating *Signal Magnitude Vector* by using the following relation:

(1) $$\text{Signal Magnitude Vector}=\sqrt{{\left|A_{x}\right|}^{2}+{\left|A_{y}\right|}^{2}+{\left|A_{z}\right|}^{2}}$$

where *A_x_*, *A_y_*, and *A_z_* represent tri-axial accelerometer signals of the *x*, *y*, and *z*-axis respectively. If the value of signal magnitude vector for a particular incident exceeds a predefined threshold value, then the algorithm primarily identifies that incident as a fall event. To make the final decision, algorithms usually depend on the next communication phase.

The processing power of SP processors has increased dramatically over the past few years. The computational power of the latest SPs has become comparable to that of former workstations [35] and, thus, even complex machine learning and statistical classification algorithms for fall detection and prevention can easily be implemented in SPs [36]. Zhao *et al.* [37] implemented three machine learning algorithms, namely C4.5, Decision Tree (DT) [38], Naïve Bayes (NB) Classifier [39] and Support Vector Machine (SVM) [40], on SPs and compared their recognition accuracy. He and Li [8] employed a combined algorithm of Fisher's Discriminant Ratio (FDR) criterion and J3 criterion [41] for fall detection. Majumder *et al.* [22] applied Hjorth mobility and complexity [42] for classifying gait and hence developed a fall prevention system. Some solutions [21,43] include external sensors and processing units, using the SP for sensing and/or communicating with the users and/or their caregivers.

#### 2.1.3. Phase 3: Communication

Depending on the sensor's responses from the first phase, preliminary detection or prediction of falls events is performed by algorithms in the second phase. Whenever a SP-based solution detects or predicts a fall event, it communicates with the user of the system and/or caregivers. Most fall detection solutions carry out this communication phase in two steps. In the first step, the system attempts to obtain feedback from the user by verifying the preliminary decision and thus improve the sensitivity of the system. The second step depends on the user's response. If the user actively rejects the suspected fall, then the system restarts. Otherwise, a notification is sent to caregivers to ask for immediate assistance. Some systems may not wait for user's feedback and will immediately convey an alert message to the caregiver [44,45]. Rather than requesting feedback, fall prevention systems generally alert the users about their imminent fall. Moreover, instead of alerting the users, fall prevention systems can also activate other assistive systems (e.g., wearable airbag [16,46–48], intelligent walker [49,50], intelligent cane [51,52], intelligent shoe [53], *etc.*) for protecting the user from the adverse effects of falling.

User's feedback can be collected automatically by analyzing the sensor's output. For example, the algorithm proposed by Sposaro and Tyson [34] generates the final decision by automatically analyzing the difference in position-data before and after the suspected fall event. Other systems demand manual feedback from the user. Requests for the user's feedback can be attempted by using the external speakers on the phone and requesting a vocal or keypad response from the user [21]. Combinations of alarm systems and graphical user interface of SPs are also used for collecting the feedback of the user [9,54]. After requesting a response from the user, the system waits for a pre-defined period (typically ≤ 1 min). If the user does not respond within that time, the system will consider the event as a fall. Fall detection systems may fail to detect a real fall event automatically. In such cases, some systems provide *help* (or *panic*) buttons and thus allow users to seek help manually [55].

Smartphone-based systems generate several types of notifications to seek help from caregivers or for forewarning the users about an imminent fall such as audible alarms [56], vibrations [22], Short Message Service (SMS) [34,43,57], Multimedia Messaging Service (MMS) [8,27], and even automatic voice calls [21,57]. E-mails and Twitter messaging have also been described [2]. Notification messages may contain information on time [27], Global Positioning System (GPS) location (coordinates) [27,29,57], and location map [2,26,58]. SP-based solutions can also support streaming of phone data from microphones and cameras for further analysis of the situation [21].

### 2.2. Taxonomy

This section presents a detailed taxonomy of SP-based fall detection and prediction systems with respect to the three different phases of operation: sense, analyze and communicate. Here we focus on the categorization of various attributes/aspects of SP-based solutions for fall detection and prevention. The aim of this taxonomy is to provide a complete reflection of the properties of existing as well as possible SP-based solutions. The correctness and completeness of the taxonomy will be reflected upon in Section 3.

**Figure 2.**
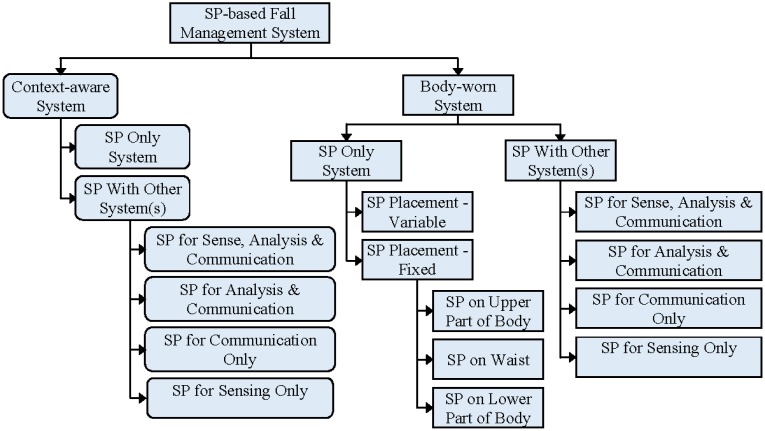
Taxonomy of smartphone-based systems based on sensing mechanism and sensor placement.

Figure 2 illustrates the taxonomy of SP-based fall detection and prevention technologies based on their sensing mechanism and sensor placement. Existing solutions are represented with rectangles, while rounded rectangles represent possible solutions that have not previously been reported to identify areas for future research. SP-based solutions can be categorized into two types: context-aware and body worn. With context-aware systems, the user should not wear any sensor or system. Sensors are placed in the surrounding and the user can move freely, but within the catchment areas of the sensors. Though, the main advantage of context-aware systems is that the person does not need to wear any special device, their operation is limited to those places where the sensors have been previously deployed [59]. No such SP-based context-aware solution has been found. All the SP-based solutions, proposed so far, are body worn systems and users are required to keep their SPs close to their body. This type of solution can be further classified according to the existence of external sensor(s)/system(s) and the placement of the SP.

**Figure 3.**
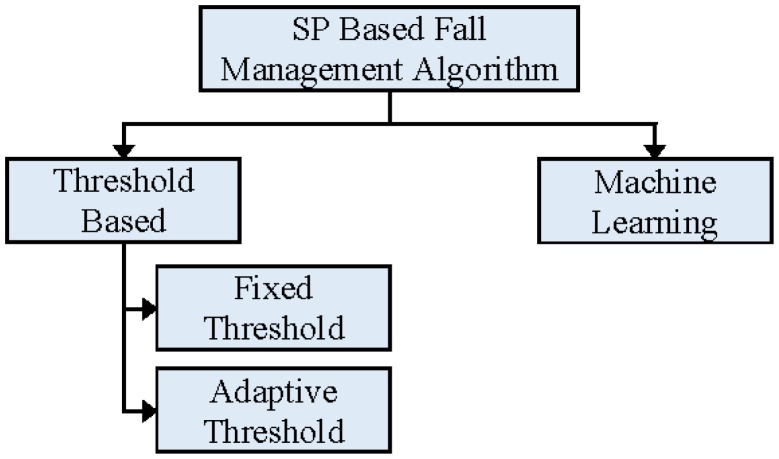
Taxonomy of smartphone based fall detection and prevention algorithms.

Smartphone-based solutions can also be categorized on the basis of algorithms used in the analysis phase. Figure 3 presents the taxonomy of SP-based fall detection and prevention algorithms. Due to the lower processing capacity and low energy storage capacity of batteries in SP compared to desktop or laptop computers, SP-based solutions mostly use TBAs for the detection or prediction of falls events. Machine learning algorithms are also attracting research interest because of the improved processing and battery capacities of newer, high-end, SPs.

**Figure 4.**
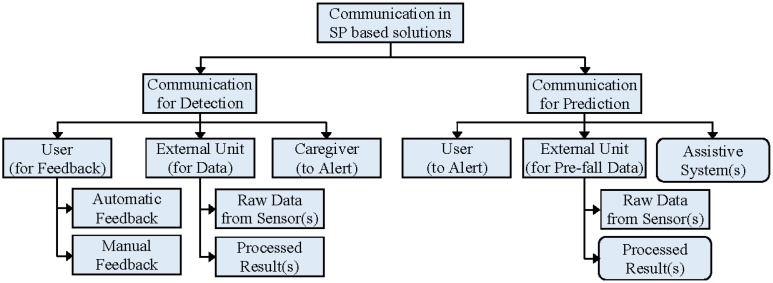
Taxonomy of communication patterns in smartphone-based fall detection and prevention systems.

Existing and potential SP-based fall detection and prevention systems communicate with the users, caregivers or assistive systems by sending alert signals, obtaining user or system feedback or activating assistive systems. The taxonomy of communication patterns in SP-based fall detection and prevention is shown in Figure 4. Rectangles and rounded rectangles hold the same meaning as in Figure 2. Detection systems communicate with the users to obtain feedback, whereas prediction systems communicate to alert them about their possible forthcoming falls. Prediction systems are only concerned with pre-fall data, but detection systems deal with pre-fall, post-fall and intermediate data. Finally, detection systems notify caregivers of fall events and ask for help, whereas prediction systems attempt to prevent impending falls with the help of other assistive systems. Some SP-based solutions require external sensing units that may or may not have built-in processors. These external units may transmit either raw data or results after primary analysis. No article has been found, that uses assistive system and/or external processing unit for implementing SP-based fall prevention solution.

## 3. Comparative Analysis

In the reviewed articles, the authors commonly report their main objective (detection/preventing), usability (sensor placement & type), the SP operating systems, algorithm novelty, efficiency (sensitivity and specificity) and notification techniques. For comparison we focused on those features, which are inevitable or have comparatively more variants. Other features have been discussed separately. This section compares existing works based on their functional and architectural properties and quantitative properties.

We included journal articles and conference proceedings published on SP-based fall detection and fall prevention. Advanced Boolean searches are conducted, with no time limit, in MDPI, IEEE Xplore, PubMed, Web of Knowledge and Google Scholar with the search condition: “Find articles with all the words {keyword1 AND keyword2} anywhere in the article”. The keyword “smartphone” is always inserted as keyword1 with any one of the other three keywords: “fall detection”, “fall prevention” and “fall prediction”. Each keyword is inserted within double quotation marks and two keywords are separated by a Boolean operator AND. Additional articles are identified from the cross-referencing from these articles. A total of 578 articles are matched our search criteria. Among these articles, 51 articles included some experimental results or pioneering investigations on SP-based solutions for fall detection and fall prevention and are selected for further review. The remaining articles were excluded as they have used these keywords for other purposes such as, use of their proposed systems, references, and examples.

### 3.1. Functional and Architectural Comparison

Common built-in sensors of recent SPs and their corresponding functions are shown in Table 1. Examples of fall detection and prevention or related solutions (SP-based or non-SP-based), which use similar dedicated sensors, are also included, to identify potential new areas for research.

#### 3.1.1. SP-Only Systems

Depending on the uses and placement of sensors the SP-based solutions are categorized into two major categories: context-aware systems and body-worn systems (see Figure 2). Table 2 summarizes and compares the important features of existing SP only systems. In this table the articles are organized chronologically.

#### 3.1.2. Smartphones with Other External Systems

Table 2 shows that most of SP-only systems demand fixed placement of SPs, but this is considered as a usability constraint, because not all people carry their mobile phones in a fixed position [31]. Moreover, sensors in SPs usually have much lower resolutions than dedicated sensors [33]. Body-worn systems can also use external sensing and processing units together with SPs to overcome these two constraints. Some of these external units are used only for sensing or measuring physical quantities [31,32]. These units will transmit raw data to the SP, and then the SP will perform feature extraction, classification and notification tasks. External units can also perform the feature extraction and classification tasks with the help of attached microcontrollers. Such units will communicate with the SP for the communication step. Moreover, these external units will minimize the computational load and wireless communication burden of the SP and reduce battery consumption. External components, which are used in various SP-based fall detection and prevention solutions, are listed in Table 3.

Features of SP-based fall detection and prevention solutions, which employ external system(s) along with SPs, are summarized in Table 4. Smartphones with other external systems can be subcategorised, based on three phases of operations, into four types as shown in Figure 2. Such solutions can utilize SP for all of the three phases of operations while employing external units for the sensing phase only. It is also possible to use SPs for only the sensing or communication phases, but such systems must use external microcontrollers for analysis. If the SP is only used for the sensing phase, then for acquiring less ambiguous signals, it is important to firmly attach the SP at a fixed position of the user's body, but not all users like to carry their SPs in a fixed location. In order to overcome this constraint, some solutions utilize SPs for both analysis and communication phase and an external sensor for the sense phase. Since the SP is mainly a communication device, using SPs for analysis phase only or for both sensing and analysis phases is not a better solution. Moreover, using SPs for sensing and communication phase is also an impractical solution, because that will demand excessive wireless communication and thus consume excessive battery power. We therefore omit the latter three options from our taxonomy and Table 4 also supports our decision.

### 3.2. Quantitative Analysis

This section presents some statistical and time series analysis based on the articles that have been compared in Tables 2 and 4. The most important feature, that is not included in these articles, is the performance or the correctness of the reviewed solutions. More than half of the articles [2,21,26,27,29,32,34,43,45,54,55,57,65,70,71,73,74,77,79,82,85,89,91,93–95] do not declare the performance/accuracy of their systems, because these articles present very preliminary investigations on SP-based fall detection and fall prevention. The remaining articles, included in Table 5, discussed the performance of their proposed solutions but there were major differences between the evaluation techniques. Moreover, their test results were obtained by analysing simulated falls events, not true falls.

The existing solutions tried to detect and classify the falls events, risk of falls and other normal ADLs accurately. Usually, the performance of such solutions is examined based on the sensitivity, specificity and total accuracy [97]. Some articles [64,87] measured the performance of their proposed systems in a different way. They used the performance parameters: precision and recall [80] Some other articles measured the accuracy of their proposed systems, simply by finding the ratio of number of correctly identified cases and the total number of cases [58,92]. Same as fall detection systems, standard approach for describing accuracy of fall prevention systems has been through sensitivity (proportion of fallers correctly classified as high fall risk) and specificity (proportion of nonfallers correctly classified as low fall risk) [98]. Table 5 summarizes the declared performances of the SP based fall detection and prevention solutions.

Fifty-one SP-based solutions are compared in Tables 2 and 4 and forty-one (80%) solutions used SP with the Android operating system. The Android platform is preferred [8,33,83] as it is an open source framework designed for mobile devices [34,78,89]. Other SP operating systems which have been used in fall detection and prevention solutions include iOS (8%) [22], Symbian OS (6%) [64] and Windows Mobile (4%) [57]. One paper (2%) did not report the SP operating system they used.

**Figure 5.**
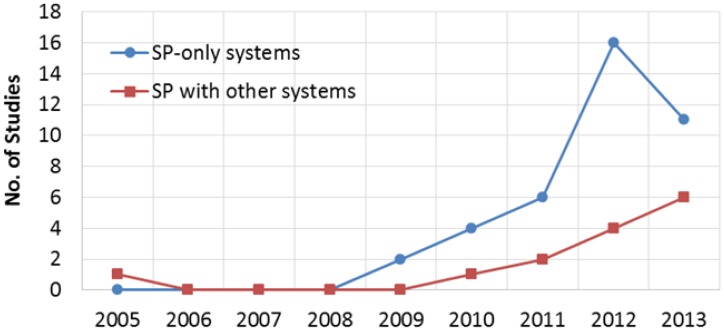
Estimation of the number of SP based fall detection and prevention studies.

The accelerometer was used in all the reviewed solutions and the GPS receiver is the second most commonly used sensor (42%) followed by the gyroscope. In addition we have performed a time series analysis on SP based fall detection and prevention solutions and the outcome is shown in Figure 5. This line chart shows a comparison of the numbers of studies on SP-only solutions with other solutions having a combination of SP and external devices. In the past few years, though the number of studies on SP-only solutions are higher than those of other SP based solutions, the use of external devices in SP based fall detection and prevention systems is increasing gradually.

## 4. Discussion

Various benefits of using the SP as a pervasive fall management system have already been discussed [28]. Despite all these benefits, SP-based systems do face some critical challenges with certain issues remaining open to further research. Based on our extensive literature review, these challenges and open issues in SP-based fall management systems have been identified. This section presents the most relevant ones.

### 4.1. Challenges

#### 4.1.1. Quality of SP Sensors

It remains doubtful whether the qualities of built-in SP sensors in existing SPs are adequate to produce fall detection and prevention systems with acceptable performance. The SP sensor that is used by all SP-only solutions is the accelerometer and the usual dynamic ranges of these built-in accelerometers are insufficient for accurate fall incident detection [31]. Acceptable dynamic ranges for accelerometers from ±4 g to ±16 g have been mentioned in previous publications (where, 1 g = 9.8 m/s^2^) [31,33,99]. Smartphones typically contain accelerometers with dynamic ranges of ±2 g or less [33], but higher dynamic ranges can be found in high-end SPs [81]. While choosing an SP for a particular application (fall detection or fall prevention) adequate attention should be paid to the quality of the sensors. Specifications of the sensors should satisfy the minimum requirements of the applications. Similar attention should be paid to all other SP sensors.

#### 4.1.2. Energy Consumption and Battery Life

A major weakness of SP-based solutions is the limited battery life of SPs. Usually the battery life of an SP in normal use is about one day [33], but no SP battery will last more than a few hours with heavy usage [36,100]. The issue of energy consumption should therefore be considered when designing an SP-based system. The energy consumption or battery life of the SP is dependent on the number of sensors used [54], data sampling frequency [28,54], data recording time [75], features of algorithm [87] and mode (backend or frontend) of operation [26]. The battery life of a particular SP (Samsung Galaxy S II) was reduced from 30 h when only one sensor was used, to 16 h if three sensors were used simultaneously [54]. Majumder *et al.* [22] showed that an iPhone, which runs a machine learning algorithm, can run for at most 3 h with a fully charged battery. The battery life is also directly proportional to the recording time and activities of user [74].

While choosing the right algorithm, care should be taken to incorporate a minimal number of features, fewer features would decrease the usage of processor and would save energy [87]. Experimental results of [26] shows that the consumption rate of the battery per hour for foreground execution mode and background execution mode are 2.5% and 2.25% respectively. However, energy saving measures could adversely affect accuracy and usability.

#### 4.1.3. SP Placement and Usability Issues

Smartphone-based fall detection and prevention systems are mostly designed for older people and individuals with neurodegenerative disorders. However, the acceptability of these solutions among older individuals has been suggested as a limiting factor [31]. People with intellectual disabilities also face great difficulty using the complicated interfaces of modern SP-based applications [101,102]. A recent study has revealed the myth that older people avoid new technologies is a fallacy [103]. Older people have been found to be willing to accept new technologies to support their independence and safety [104]. The older person may also prefer to have a single phone with self-contained fall detection functionality than to wear a separate fall detection device [22].

As mentioned earlier, all SP-only solutions use the accelerometer as a sensor which requires fixed placement of the SP. Various fixed positions on the body have been proposed, such as: the shirt pocket [73], waist [44] and trouser pocket [70]. This requirement limits the usability of SP-based solutions because not everyone caries their SP in a fixed position [31] and it may be difficult to convince them to do so [105]. In order to overcome this obstacle, researchers have proposed the use of external body-worn sensors in combination with SPs. This solution is also not accepted universally because these external devices expose the frailty of the user [33] and many users forget to put on such external devices [106]. Therefore, while designing new SP based solution, SP placement and usability issue should be handled carefully.

### 4.2. Open Issues

#### 4.2.1. SP Based Context-Aware Fall Detection and Prevention

Context-aware fall detection and prevention systems use sensors deployed in the environment to detect or predict falls. The main advantage of such systems is that the user does not need to wear any special device on his or her body [59]. Due to this advantage, several context-aware fall detection and prevention solutions using various conventional external systems have been proposed [62,69,107–109]. No previous report has been found in our literature search on SP-based context-aware solutions. Existing SP based solutions are body-worn type, but at home, users usually do not carry SPs on their bodies, so those SP based solutions are not suitable for home environments. Users should depend on separate conventional context-aware solutions at home. In this context, single SP based solution having both body-worn and context-aware modes of operations would be a better alternative to using separate solutions for indoor and outdoor protection. Such a SP-based solution may run in body-worn mode and context-aware mode when the user goes outside and comes back home, respectively. Automatic switching between two modes of operations is also possible.

The taxonomy of such SP-based systems is shown in Figure 2. Han *et al.* [110] have proposed a multimodal approach which utilizes the set of embedded sensors (accelerometer, audio tool, GPS, Wi-Fi, *etc.*) on smartphones in order to recognize eight different user contexts, such as walking, jogging, riding on a bus, or taking the subway. Although this system does not recognize fall events, it provides feasible support for SP-based context-aware fall detection and prevention solution. The sensors that are used frequently in traditional context-aware systems are cameras, infrared sensors, microphones and pressure sensors. Most of these sensors are also available in modern SPs. Moreover the computational and processing capacities of SPs are continuously improving. Therefore it is highly possible to use SPs for context-aware fall detection and prevention. For small monitoring area, such as a single room, context-aware system may require a single sensor. Such single sensor (e.g., camera) based context-aware system can be completely replaced with SP-only context-aware system. In that case, SP should be kept at the place (e.g., wall mounted holder) of that sensor during its context-aware mode of operation. It should be noted that we have proposed this novel concept of SP-based context-aware system based on our own observations.

#### 4.2.2. Smartphones with Other Assistive Devices for Fall Prevention

Smartphone-based fall prevention is comparatively less explored with respect to SP-based fall detection. Among 51 reviewed articles only five articles [22,30,65,74,95] reported or evaluated fall prediction solutions and two articles [9,54] dealt with both fall detection and prediction. All previously reported solutions attempted to prevent falls by early prediction and alerting the user for imminent falls. Previous reports have only described fall prediction systems, but a working SP-based prevention system linked to assisted devices has not yet been achieved. Wu and Xue [16] proposed a pocket PC-based fall prevention system. This system can detect falls events at least 70 ms before the impact and activate an inflatable hip pad for preventing fall-related hip fractures. Since SPs can be easily substituted for Pocket PCs, this system demonstrates that SP-based fall prevention systems can be designed with the help of other assistive devices like airbags or inflatable hip pads.

#### 4.2.3. Real-Life Falls Analysis

Falls in individuals occur relatively infrequently in real-life even in individuals with increased susceptibility to falls [111]. Therefore, only two of the SP-based solutions reviewed had evaluated their system in real-life falls [31,94]. The remaining articles only evaluated their system within simulated falls situations. Klenk *et al.* [112] demonstrated that simulated falls and real-life falls differ in terms of acceleration magnitude and dynamics. Consequently, the performances measured on simulated falls situations are considered inadequate for robust testing of fall detection and prevention systems [113]. Evaluation of SP-based fall detection and prevention systems in real-life conditions should therefore be considered a vital area for future research.

## 5. Conclusions

In this paper we have comprehensively evaluated the existing literature on SP-based solutions for fall detection and prevention. Built-in inertial sensors, open source operating systems, state-of-the-art wireless connectivity and universal social acceptance make SP a very good alternative to conventional dedicated fall detection and prevention tools. However, the performance and usability of current systems remain limited by the relatively lower quality of in-built sensors such as accelerometers in existing SP devices, as well as the need to wear the SP in a fixed position for SP-only solutions. The addition of component parts or additional systems may resolve these issues, but reduces the attractiveness of SP-based solutions. Future research should be aimed at context-aware fall detection and prevention systems which do not require the device to be worn as well as assessment of fall detection and prevention systems in real-life situations.

## Figures and Tables

**Table 1.** Smartphone built-in sensors and their uses.

**Built-in Sensors of SP**	**Usual Use in SP**	**Use in Fall Detection & Prevention**
Accelerometer	Senses the changes in orientation of SP and adjusts the viewing angle accordingly.	[60]
Gyroscope	Detects angular momentum (roll, pitch and yaw); facilitates game.	[60]
Magnetometer	Senses the Earth's magnetic field; works as a digital compass.	[60]
Barometer	Measures atmospheric pressure; facilitates weather widgets.	[61]
Image Sensor	Provides still picture and video capturing facilities.	[62]
Microphone	Sound capture.	[63]
Wi-Fi sensor	Facilitates wireless communication through Wi-Fi.	[64]
Bluetooth Sensor	Facilitates wireless communication through Bluetooth.	[60]
Location sensors (GPS)	Targets or navigates by map or picture with the help of GPS satellites.	[2]
Temperature Sensor	Measures temperature; facilitates weather widgets.	[65]
Humidity Sensor	Measures humidity; facilitates weather widgets.	[65]
Ambient Light Sensor	Adjusts the display brightness.	[66]
Proximity Sensor	Detects how close our SP's screen is to our body.	[67]
Touch Sensor	Helps to operate the SP through touching.	-
NFC Sensor	Establishes communication between similar device by touching or bringing them into proximity.	[68]
Infrared Sensor	Can sense temperature.	[69]
Back-Illuminated sensor	Adjust the light captured while capturing a photograph.	-

**Table 2.** Comparison of smartphone-only fall detection and prevention systems.

**Year**	**Article**	**Objective**	**SP Position**	**Sensor(s)**	**Algorithm(s)**	**Alerting Feature(s)**
2009	[34]	Detection	Any	Accelerometer	TBA (Adaptive: depends on user provided parameters)	SMS (time, GPS coordinates, password for activating bidirectional voice call).
	[70]	Detection	Trouser Pocket	Accelerometer	TBA (Fixed)	SMS, voice call, vibration, sound.
2010	[28]	Detection	Chest, Waist, Thigh	Accelerometer & gyroscope	TBA (Fixed)	Sound alarm, voice call.
	[2]	Detection	Trouser Pocket	Accelerometer	Discrete Wavelet Transform (DWT)	SMS (GPS coordinates), email (Google Map), twitter messages.
	[56]	Detection	Chest, Waist, Thigh	Accelerometer	TBA (Fixed)	Audible alarm, voice call.
	[37]	Detection	Waist	Accelerometer	C4.5 DT, NB and SVM	SMS
2011	[9]	Detection	Waist	Accelerometer	TBA (Fixed)	E-mail and/or SMS.
	[44]	Detection	Waist	Accelerometer	TBA (Fixed)	SMS (date, time, location)
	[71]	Detection	Pocket	Accelerometer	TBA (Fixed)	SMS (name, time, GPS coordinates, street address)
	[72]	Detection	Hand, Shirt or Trouser Pocket	Accelerometer & gyroscope	TBA (Fixed), One-Class SVM	Not found
	[45]	Detection	Not found	Accelerometer	TBA (Fixed)	Audible alarm, SMS (GPS coordinates), voice call (manual), remote server draws help path
	[73]	Detection	Shirt Pocket	Accelerometer	TBA (Fixed)	SMS
2012	[26]	Detection	Waist	Accelerometer	TBA (Fixed)	SMS (time, GPS data), draw help path
	[27]	Detection	Waist	Accelerometer	TBA (Fixed), Median filter attenuate noise	MMS (time, map of suspected fall location, and GPS coordinate)
	[31]	Detection	Waist	Accelerometer	TBA (Fixed), ANN ^1^ based pattern classifier	Notification contains GPS coordinates.
	[54]	uFall for Detection, uTUG for Prevention	Waist	Accelerometer, Gyroscope	TBA (Fixed)	E-mail or SMS, recorded signals are sent to remote server, audio cue (for uTUG)
2012	[74]	Prevention (GUG)	Waist	Accelerometer	Segmentation, filtering, dispersion measures calculation	Not found
	[75]	Detection	Waist (Back)	Accelerometer	SVM, SMLR ^2^ in SP, NB, DT, KNN ^3^ in PC	Not found
	[76]	Detection	Shirt or Trouser Pocket	Accelerometer	TBA (Considers axis wise data separately)	Not found
	[77]	Detection	Shirt Pocket	Accelerometer	TBA (Adaptive)	Not found
	[78]	Detection	Shirt Pocket	Accelerometer	TBA (Adaptive)	Text message
	[79]	Detection	Waist	Accelerometer	TBA (Fixed), Median Filter,	MMS (time, GPS coordinate, Google map)
	[80]	Detection	Trouser Pocket	Accelerometer	SVM classifier	Vibration, sound alarm, SMS (time, location, & health information)
	[64]	Detection	Waist	Accelerometer, Wi-Fi module	DT Classifier, location estimation using RSSI ^4^	SMS (name, time, location)
	[81]	Detection	Hand, Pocket, waist	Accelerometer, Gyroscope	Semi-supervised learning	Not found
	[82]	Detection	Not found	Accelerometer, Gyroscope	Not found	SMS (location),
	[83]	Detection	Chest, Waist, Thigh	Accelerometer	TBA (Adjusted based on user's profile)	SMS
	[84]	Detection	Hand, Pocket	Accelerometer, Gyroscope	TBA (Fixed)	Not found
2013	[57]	Detection	Trouser Pocket	Accelerometer	TBA (Fixed)	SMS (date, time, GPS data), voice call, vibration, sound.
	[8]	Detection	Chest	Accelerometer, Gyroscope, & Magnetometer	Fisher's discriminant ratio and *J*3 criterion	MMS (time, map of suspected fall location, GPS coordinate)
	[22]	Prevention	Trouser Pocket	Accelerometer & Gyroscope	C4.5 DT classifier, Hjorth mobility and complexity [42]	Alert the user about imminent fall by using message & vibration.
	[33]	Detection	Waist	Accelerometer	TBA (Fixed)	SMS, voice call, others: twitter, email, Facebook.
	[55]	Detection	Not found	Accelerometer	TBA (Fixed)	SP trigger PC via Wi-Fi, PC send alert via SMS, emails or/and voice calls
	[58]	Detection	Waist	Accelerometer	TBA (Fixed)	SMS (time, GPS data), draw help path
	[85]	Detection	Not found	Accelerometer	TBA (Fixed)	Not found
	[86]	Detection	(User's height 164 cm)	Accelerometer	TBA (Fixed)	Server displays current states and triggers an alarm
	[87]	Detection	Trouser Pocket	Accelerometer	OneRAttributeEval, ReliefFAttributeEval SVMAttributeEval, K* [88], C4.5, NB	SMS (GPS coordinate)
	[89]	Detection (Free Fall)	Not found	Accelerometer	Displacement based algorithms	SMS (GPS coordinate)
	[90]	Detection	Waist	Accelerometer	TBA (Fixed)	SMS

1. Artificial Neural Network;

2. Sparse Multinomial Logistic Regression (SMLR);

3. k-Nearest Neighbours (KNN);

4. Received Signal Strength Indication.

**Table 3.** External components, used in SP-based fall detection and prevention solutions.

**Component Name**	**Features**	**Used In**
SensorTag (TI)	Temperature, Humidity, & Pressure Sensor, Accelerometer, Gyroscope, Magnetometer, Bluetooth, 8051 Microcontroller	[43]
Shimmer2 (Shimmer)	Accelerometer, 802.15.4 standard Radio, Bluetooth Module, MSP430 Microcontroller	[31]
GPSADXL	2-axis Accelerometer (Two), GPS Module	[21]
BlueGiga WRAP	Bluetooth RS-232 cable replacer	[21]
Camera	Video Camera	[29]
X6-2 Mini (Gulf Coast)	Accelerometer	[75]
ADXL335	Accelerometer	[91]
ADXL345	Accelerometer	[92]
BC5 (CSR Inc.)	Bluetooth Module	[92]
EZ430 Chronos (TI)	Accelerometer, Pressure, Temperature & Battery Voltage Sensor, Bluetooth Module, MSP430 Microcontroller	[93]
CC1111 (TI)	USB RF Access Point	[93]
LIS344ALH (STMicro)	Accelerometer	[94]
BlueGiga WT12	Bluetooth Module	[94]
XBee RF (Digi)	ZigBee Module	[94]
XU-Z11 (Digi)	USB to ZigBee Adaptor	[94]
XR-Z14-CW1P2 (Digi)	ZigBee Wall Router	[94]
Bed Presence (Ibernex)	Detects the absence of user on bed	[94]
PIC24F (Microchip)	Microcontroller	[65,94]
Piezoresistive sensors	Can measure mechanical stress	[30]
Arduino	Microcontroller	[30,91]
WiFly Shield	Able to connect to 802.11b/g wireless networks	[30]
NODE (Variable Tech)	Accelerometer, Gyroscope, Magnetometer, Bluetooth Module	[95]

**Table 4.** Fall detection and prevention systems using smartphone and other external units.

**Year**	**Article**	**Objective** *	**Sensor(s)**	**SP Position**	**External Sensor's Position**	**SP—External Unit Connectivity**	**Analysis Unit**	**Algorithm(s)**
2005	[21]	D	SP camera, External accelerometer	Any	Waist	Bluetooth	External PC	Not found
2010	[28]	D	SP accelerometer, gyroscope & magnetometer, Several external magnets (35 mT)	Trouser right (left) Pocket	Just above left (right) knee	Magnetic Field	SP	TBA (Fixed), Hausdorff distance
2011	[32]	D	External accelerometer & gyroscope	Any	Waist, left & right ankle	ZigBee	SP	Center of gravity clustering algorithm
	[96]	D	SP accelerometer & gyroscope	Not found	Chest, Finger tip	Bluetooth	External PC	TBA (Fixed)
2012	[31]	D	External accelerometer	Any	Waist	Bluetooth	SP	ANN Based Pattern Classifier
	[91]	D	External accelerometer	Any	Chest	Bluetooth	External Arduino Board	TBA (Fixed)
	[92]	D	External accelerometer	Not found	Chest/Waist	Bluetooth	SP	TBA & Binary DT
	[65]	P	External bend, temperature & humidity sensor, accelerometer, gyroscope	Not found	Shoe-Sole	Bluetooth	SP	SVM, Fast ANN & TBA
2013	[29]	D	SP accelerometer & GPS receiver, External video camera	Chest	Wall mounted	Client/Server network	SP & Network PC	Both TBA & machine learning
	[43]	D	SP GPS Module, External accelerometer	Any	Torso	Bluetooth	External Unit	Not found
	[93]	D	External accelerometer	Any	Wrist	Bluetooth	External PC	TBA (Fixed)
	[94]	D	External accelerometer, gyroscope, bed presence sensor	Any	Waist	Bluetooth	External Unit	Not found
	[30]	P	SP accelerometer & gyroscope, External pressure sensor (4 units),	Pocket or Hand	Shoe-Sole	Wi-Fi	SP	Hjorth mobility and complexity, Energy Integral
	[95]	P	External accelerometer & gyroscope (two sets)	Not found	Chest and Arm	Bluetooth	SP	TBA (Fixed)

* “D” represents *Detection* and “P” represents *Prevention*.

**Table 5.** Declared performances of the SP based fall detection and prevention solutions.

**Article**	**Objective**	**Declared Performance**
[8]	Detection	The total classification accuracy is 95.03% (accuracies for static, transitions, dynamic, and falls are 98.75%, 94.625%, 91.8%, and 97.63%, respectively)
[9]	Detection	Both specificity and sensitivity are 100%, except the case when fall dynamics is completely in the vertical direction
[22]	Prevention	99.8% accuracy in gait abnormality detection
[28]	Detection	Average of false negative values is 2.13% and the false positive value is 7.7%
[30]	Prevention	97.2% accuracy in gait abnormality detection
[31]	Detection	Obtained 100% sensitivity, specificity, and accuracy
[33]	Detection	Sensitivity 83.33% and a specificity 100%
[44]	Detection	Specificity and sensitivity are 81% and 77% respectively
[56]	Detection	Waist is the best position to attach the phone and gives average false negative value of 2.67% and false positive value of 8.7%.
[58]	Detection	Accuracy 94% (50 samples for the test and 47 of these samples are correct)
[64]	Detection	Precision & Recall (respectively) for DT: 100% & 75.8%; for SVM: 99.81% & 75.43%; for NB: 98.67% & 73.20%
[37]	Detection	Accuracy for DT is 98.85%, for SVM is 86.47%, and for NB is 87.78%
[72]	Detection	Accuracies are 75% (while typing SMS), 87.5% (while listening), 77.9412% (SP in chest pocket) and 84.2857% (SP in pants pocket)
[75]	Detection	Identify falls with 98% accuracy and classify the type of falls with 99% accuracy
[76]	Detection	Average sensitivity & specificity are 97% & 100% respectively
[78]	Detection	Sensitivity 92.75% and specificity 86.75% (for adaptive TBA)
[80]	Detection	Average recall is 90% and precision is 95.7%
[81]	Detection	Sensitivity 85.3% and specificity 90.5%
[83]	Detection	72.22% sensitivity and 73.78% specificity
[84]	Detection	Sensitivity 80%, specificity 96.25% and accuracy is 85%
[86]	Detection	Accuracy is 86% in lying and 100% in falling
[87]	Detection	Precision & Recall (respectively) for NB: 83.8% & 82.0%; for J48 DT: 88.2% & 88.3% for K-Star: 88.9% & 88.6%
[90]	Detection	90% specificity, 100% sensitivity and 94% accuracy
[92]	Detection	Overall accuracy of 92%
[96]	Detection	Falls (active) accuracy 95.2%, Falls (inactive) accuracy 95.7%
